# Efficacy and safety of MuShengshu in the treatment of mild-to-moderate atopic dermatitis: protocol for a randomized, double-blind, placebo-controlled trial

**DOI:** 10.3389/fmed.2026.1845534

**Published:** 2026-05-21

**Authors:** Xiangjin Gao, Ruiqi Cai, Jinrong Lu, Zhen Duan, Quanruo Xu, Xiuqi Zhang, Fanlingzi Shen, Rui Zhang, Bin Li, Ruiping Wang

**Affiliations:** 1 Clinical Research Center, Shanghai Skin Disease Hospital, Tongji University, Shanghai, China; 2 School of Public Health, Shanghai University of Traditional Chinese Medicine, Shanghai, China; 3 School of Medicine, Tongji University, Shanghai, China

**Keywords:** atopic dermatitis, clinical trial protocol, MuShengshu, randomized controlled trial, traditional Chinese herbal bath solutions

## Abstract

**Background:**

Atopic dermatitis (AD) is a prevalent chronic and recurrent inflammatory skin disease. Guidelines for the treatment and care of AD suggest that appropriate bathing can facilitate the repair of skin lesions. MuShengshu is a weakly acidic bath solution that contains traditional Chinese medicine ingredients. Previous studies indicate its potential to alleviate pruritus and promote the healing of skin lesions in patients with dermatosis. However, high-quality evidence is still limited.

**Objectives:**

This randomized, double-blind, placebo-controlled clinical trial aims to assess the efficacy and safety of MuShengshu (MSS) combined with foundational therapies in patients with mild-to-moderate AD.

**Methods:**

A total of 66 patients with mild-to-moderate AD will be enrolled at the Shanghai Skin Disease Hospital from August 2025 to June 2026. Patients will be randomly assigned in a 2:1 ratio to either the treatment group (MSS combined with foundational therapies, *n* = 44) or the control group (MSS placebo combined with foundational therapies, *n* = 22). Patients will receive MSS or MSS placebo once daily, for 4 months. Meanwhile, the foundational therapies include desonide cream for mild AD and triamcinolone acetonide cream for moderate AD, both of which will be applied twice daily for 2 weeks. Additionally, all patients will receive urea ointment twice daily for 4 weeks. The primary outcome indicator is EASI_50_, defined as the proportion of patients achieving ≥50% improvement in the Eczema Area and Severity Index (EASI) score at week 4 from baseline. The secondary outcome indicators include the EASI, Visual Analogue Scale (VAS), Investigator’s Global Assessment (IGA), and Dermatology Life Quality Index (DLQI), which will be assessed at baseline (week 0), weeks 1, 2, 4, and 16. In this study, statistical analyses will be conducted using SAS 9.4 software, with statistical significance being defined as a two-tailed *α* level of 0.05.

**Results:**

In this study, ethical approval was granted in March 2025, with registration completed in the International Traditional Medicine Clinical Trial Registry in June 2025. Recruitment of participants began in August 2025 and is anticipated to completed by June 2026. Data analysis is set to start in August 2026, with preliminary trial results expected to be submitted for peer-reviewed publication by December 2026.

**Conclusion:**

Findings in this study are expected to provide evidence for the incorporation of traditional Chinese herbal bath solutions into the comprehensive management of AD.

**Clinical trial registration:**

https://www.chictr.org.cn/, Identifier ITMCTR2025001206.

## Introduction

Atopic dermatitis (AD) is a prevalent chronic and recurrent inflammatory skin disease characterized by intense itching, recurrent attacks, dry skin, and typical eczematous lesions (1). The pathogenesis of AD is complex, involving a multifactorial interplay of genetic, immunological, and environmental factors (2). Beyond its cutaneous manifestations, AD profoundly impacts patients’ quality of life, contributing to sleep disturbances, cognitive impairments, and an increased risk of anxiety and depression (3). The incidence of AD has been rising globally, particularly among the pediatric population (4). AD is associated with a considerable disease burden, representing the highest impact among all non-fatal skin conditions (5).

In recent years, numerous treatment options for AD have been developed, including emollients, corticosteroids, calcineurin inhibitors, antihistamines, and biologics (6–8). However, current therapeutic options still have limitations, such as side effects associated with long-term use of potent corticosteroids, insufficient efficacy or frequent recurrences in some patients, and concerns about the safety and applicability of systemic therapies (9–11). Therefore, the development of new safe, effective, and easily manageable treatment options remains an important clinical challenge with substantial societal implications.

The guidelines for AD treatment and care suggest that appropriate bathing facilitates the repair of skin lesions (12, 13). Previous studies indicate that the development of AD is closely associated with impaired skin barrier function, which compromises the skin’s ability to retain moisture and defend against external irritants (14). The pH value of skin surface in healthy individuals typically ranges from 4.5 to 6.5, establishing a weakly acidic environment crucial for maintaining the integrity of the skin barrier and defending against pathogenic microorganisms (15). However, currently many commercial cleaning and skincare products are alkaline. The alkalinity can disrupt the skin’s acid–base balance, potentially increase the risk of developing or exacerbating chronic skin conditions (16). So prolonged use of such products may adversely affect individuals with dermatological issues. In contrast, traditional Chinese herbal bath solutions offer multifaceted therapeutic benefits. Formulated to restore the skin barrier and address specific dermatological disorders, these solutions also provide essential cleansing and nourishment. This synergistic approach not only aims to shorten treatment duration but also helps reduce the likelihood of recurrence (17).

MuShengshu (MSS) is a weakly acidic bath solution that contains traditional Chinese medicine ingredients. This herbal bath solution is exclusively developed, extracted, and produced by Guizhou Langyoutang Pharmaceutical Co., Ltd. Its core botanical ingredients include Cortex Dictamni, Radix Sophorae Flavescentis, Fructus Kochiae, Rhizoma Smilacis Glabrae, Isatis Root, Golden Cypress, and Agastache Rugosa. Studies indicate that MSS solution aligns with the natural pH of human skin, thereby effectively preserve the integrity of the skin barrier (18). Previous studies indicate its potential to alleviate pruritus and promote the healing of skin lesions in patients with dermatosis (19). However, there is a lack of high-quality evidence-based medical data to support these findings. We implement this randomized, double-blind, placebo-controlled clinical trial to evaluate the efficacy and safety of MSS in patients with AD. We hypothesize that MSS combined with foundational therapies will contribute to symptom relief in patients with mild-to-moderate AD. To our knowledge, this is the first trial to evaluate the efficacy and safety of a traditional Chinese herbal bath solution as an adjunct to standard therapy in mild-to-moderate AD. The results are expected to provide robust evidence for its incorporation as an adjunctive treatment in clinical practice, thereby facilitating long-term management and reducing the disease burden of AD.

## Methods

### Study design

**Figure 1:**
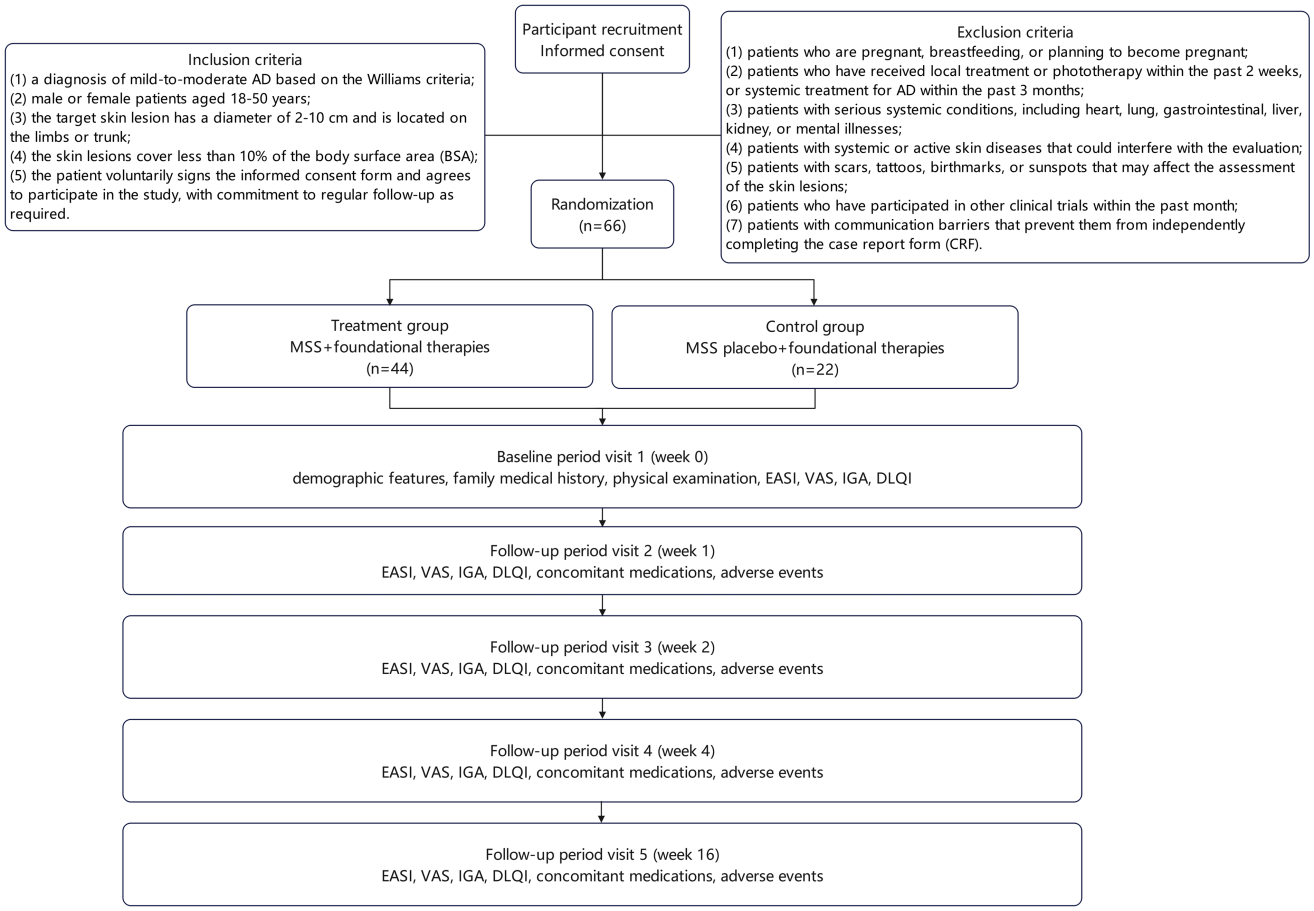
Clinical trial flowchart.

This randomized, double-blind, placebo-controlled trial is designed to evaluate the efficacy of MSS combined with foundational therapies in improving skin lesions and relieving pruritus in patients with mild-to-moderate atopic dermatitis (AD). The study protocol adheres to the SPIRIT (Standard Protocol Items: Recommendations for Interventional Trials) guidelines (20). A total of 66 patients with mild-to-moderate AD will be enrolled at the Shanghai Skin Disease Hospital during August 2025 and June 2026. Patients will be randomly assigned in a 2:1 ratio to either the treatment group (MSS combined with foundational therapies, *n* = 44) or control group (MSS placebo combined with foundational therapies, *n* = 22). The study flowchart is shown in Figure 1.

### Inclusion criteria

The inclusion criteria for patients with mild-to-moderate AD are as follows: (1) a diagnosis of mild-to-moderate AD based on the Williams criteria (Guideline for Primary Care of Atopic Dermatitis, 2022 edition (21)); (2) male or female patients aged 18–50 years; (3) the target skin lesion has a diameter of 2–10 cm and is mainly located on the limbs or trunk; (4) the skin lesions cover less than 10% of the body surface area (BSA); and (5) patient voluntarily signs the informed consent form and agrees to participate in the study, with commitment to regular follow-up as required.

### Exclusion criteria

The exclusion criteria are as follows: (1) patients who are pregnant, breastfeeding, or planning to become pregnant; (2) patients who have received local treatment or phototherapy within the past 2 weeks, or have received systemic treatment for AD within the past 3 months; (3) patients with serious systemic conditions, including heart, lung, liver, kidney, gastrointestinal or mental illnesses; (4) patients with systemic or active skin diseases that could interfere with the evaluation; (5) patients with scars, tattoos, birthmarks, or sunspots that may affect the assessment of skin lesions; (6) patients who have participated in other clinical trials within the past month; and (7) patients with communication barriers that prevent them from independently completing the case report form (CRF).

### Sample size

The sample size was calculated based on the EASI_50_ achievement rate at week 4, which was defined as the proportion of patients who achieved ≥50% improvement in Eczema Area and Severity Index (EASI) score from baseline to week 4. According to preliminary observation data, we assume that the EASI_50_ achievement rate at week 4 would be 85% in the treatment group and 50% in the control group. Based on a 2:1 randomization ratio, the sample size is calculated with PASS 15.0 software. Setting a two-tailed *α* level at 0.05 and statistical power at 80%, the calculation yields a requirement of 40 patients for the treatment group and 20 for the control group. Considering a 10% dropout rate, the final enrollment target is set at 44 patients for the treatment group and 22 patients for the control group. The specific formula for calculating sample size was as follows:

$$n_{2}=\frac{\frac{p_{1}\times (1-p_{1})}{k}+p_{2}\times (1-p_{2})}{{\left(p_{1}-p_{2}\right)}^{2}}\times {\left(\mu_{\alpha /2}+\mu_{\beta }\right)}^{2},n_{1}=k\times n_{2}$$

### Randomization

In this study, stratified randomization will be employed, with stratification factors including patient gender (male, female) and disease severity (mild, moderate). A total of 66 patients with mild-to-moderate atopic dermatitis (AD) will be randomly assigned to either the treatment group or the control group in a 2:1 ratio, based on these stratification factors. Randomization will be performed by an independent statistician using the clinical research support system to generate 66 random numbers. Allocation concealment will be ensured through the use of sequentially numbered, opaque envelopes, which will be securely stored with restricted access at the clinical research center of Shanghai Skin Disease Hospital.

### Blinding

In this study, both of patients with mild-to-moderate AD and dermatologists will be blinded to the treatment allocation. Additionally, outcome assessors, data collectors, and statisticians responsible for data analysis will also remain unaware of the group assignments throughout the trial period to prevent potential information bias.

### Interventions

Patients in the treatment group will take a daily bath using MSS for a period of 4 months. To ensure the proper application of MSS, dermatologists will advise patients to first moisten their entire body with warm water, then apply 8 g of the product (4 g per press) to the skin surface. Massage gently for 3 to 5 min to ensure full absorption, and followed by rinsing with warm water. Patients in the control group will also take a daily bath using MSS placebo for a period of 4 months. The MSS placebo was formulated to be devoid of any Chinese herbal constituents, containing only the base excipients. It was manufactured by the same entity to guarantee perfect consistency with the active treatment in all physical attributes—appearance, weight, color, odor, and packaging. The guidance and application of MSS placebo in the control group will be identical to that for the treatment group.

In this study, both of patients in the treatment group and control group will receive the same foundational therapeutic regimen. The foundational therapies include desonide cream for mild AD and triamcinolone acetonide cream for moderate AD, both of which will be applied twice daily for 2 weeks. Additionally, all patients will receive urea ointment twice daily for 4 weeks. In this study, no additional treatment is permitted, and emergency interventions require documentation in detail and will result in the withdrawal of participation.

### Outcomes

#### Primary outcome

The primary outcome indicator in this study is the EASI_50_ achievement rate at week 4. The EASI_50_ achievement rate is defined as proportion of patients achieving ≥50% improvement in EASI score from baseline to week 4.

The EASI score encompasses two main components: the affected body surface area (BSA) score (A) and clinical features score (B) for areas including the head and neck, trunk, upper limbs, and lower limbs (22). Component (A) evaluates the percentage of affected BSA for each area, which is scored on a scale of 0 to 6, as follows: 0 (0%), 1 (<10%), 2 (10–19%), 3 (20–49%), 4 (50–69%), 5 (70–89%), and 6 (90–100%). Component (B) assesses four critical clinical features: erythema (E), edema/papules/infiltration (I), excoriation (Ex), and lichenification (L). Each feature is scored based on severity, ranging from 0 to 3 points (0 = none, 1 = mild, 2 = moderate, 3 = severe). The overall EASI score is calculated by multiplying the product of (A) and (B) by regional weighted coefficients (ranging from 0.1 to 0.4) and summing these values across all regions. The total EASI score ranges from 0 to 72 points, with higher scores indicating greater severity. Based on the EASI score, the skin lesion condition of AD patients is categorized as mild (0–7 points), moderate (8–21 points), or severe (>21 points). In this study, the EASI score will be assessed at baseline (week 0), and at weeks 1, 2, 4, and 16.

#### Secondary outcomes

The secondary outcome indicators include the EASI, Visual Analogue Scale (VAS), Investigator’s Global Assessment (IGA), and Dermatology Life Quality Index (DLQI) (23, 24). These indicators will be assessed at baseline (week 0) and subsequently at weeks 1, 2, 4, and 16. A detailed study schedule is presented in Table 1, which specifies the evaluation time points for each measure.

**Table 1:** Study schedule of the MuShengshu clinical trial (16 weeks).

Indicator	Baseline	Follow-up			
	Visit 1 (week 0)	Visit 2 (week 1)	Visit 3 (week 2)	Visit 4 (week 4)	Visit 5 (week 16)
Informed consent	✓	–^a^	–	–	–
Demographic features	✓	–	–	–	–
Family medical history	✓	–	–	–	–
Physical examination	✓	–	–	–	–
Inclusion/exclusion criteria check	✓	–	–	–	–
Randomization	✓	–	–	–	–
EASI^b^	✓	✓	✓	✓	✓
VAS^c^	✓	✓	✓	✓	✓
IGA^d^	✓	✓	✓	✓	✓
DLQI^e^	✓	✓	✓	✓	✓
Concomitant medications	–	✓	✓	✓	✓
Adverse events	–	✓	✓	✓	✓

a Not applicable.

b EASI: Eczema Area and Severity Index.

c VAS: Visual Analogue Scale.

d IGA: Investigator’s Global Assessment.

e DLQI: Dermatology Life Quality Index.

### VAS

Pruritus severity in patients with mild-to-moderate AD will be assessed using the validated VAS (25). This method typically employs a 10-centimeter linear scale, with one end labeled “0 points” for “no pruritus” and the other end labeled “10 points” for “severe pruritus.” Patients mark the scale according to their pruritus perception, and evaluators determine the score by measuring the distance from the marked point to the “no pruritus” end. During each visit, participants will evaluate their peak pruritus severity over the preceding 24 h using the VAS.

### IGA

The IGA is a validated 6-point ordinal scale designed for the rapid evaluation of disease severity, with higher scores indicating greater severity (26). Dermatologists utilize specific criteria to assess key characteristics of skin lesions, as follows: 0 = clear (no erythema); 1 = almost clear (minimal erythema with barely perceptible edema or papules); 2 = mild (mild erythema with palpable edema or papules); 3 = moderate (moderate erythema with noticeable edema or papules); 4 = severe (severe erythema with marked edema or papules); and 5 = very severe (confluent erythema with significant edema).

### DLQI

The DLQI is a validated tool that systematically assesses impairment in health-related quality of life across six domains over the previous week (27). These domains include physiological responses, psychological well-being, family life, interpersonal relationships, work limitations, and social activities. Each of the 10 items is rated on a 4-point severity scale (0 = none, 1 = mild, 2 = severe, 3 = very severe), and the total DLQI score is derived by summing the scores for all items, resulting in a range from 0 to 30, where higher scores indicate greater impairment in quality of life.

### Adverse events

In this study, patients with mild to moderate AD will be made aware of any potential risks associated with the study prior to signing the informed consent form. Adverse events are defined as any unintended signs, symptoms, or diseases that occur following treatment and are not necessarily linked to the intervention. All adverse events will be documented accurately in the CRF. Additionally, dermatologists will provide appropriate treatment for any adverse events encountered, and both the adherence of AD patients and the occurrence of adverse events will be recorded.

### Withdraw and dropout

In accordance with the Declaration of Helsinki, all patients will be treated with respect and have the right to withdraw from the study at any time and for any reason. Patients’ personal information will be collected and kept confidential. If a patient chooses to withdraw from the study, the reason will be documented in the CRF.

### Data collection and management

Trained dermatologists will gather data utilizing a standardized CRF, which consists of five sections: (1) demographic information, including age, gender, and census registration; (2) family medical history related to AD; (3) physical examination data such as height, weight, and body mass index (BMI); (4) baseline (week 0) and follow-up scores for EASI, VAS, IGA, and DLQI at weeks 1, 2, 4, and 16; and (5) information on concomitant medications and any adverse events.

The database will be developed using Epidata 3.1 software, featuring comprehensive validation rules. Data for all 66 participants will be input using a double data entry method conducted by two independent operators. Consistency checks between the two datasets will help identify discrepancies, which will then be resolved by cross-referencing with the original CRF until complete concordance is achieved through iterative verification.

### Statistical analysis

In this study, data analysis will be conducted using the SAS 9.4 statistical software. Normally distributed quantitative variables will be summarized as mean and standard deviation (SD), while skewed distributions will be represented by the median and interquartile range (IQR). For inter-group comparisons of quantitative variables with normal distribution, the Student *t*-test will be utilized, whereas the Wilcoxon rank-sum test will be applied for skewed quantitative data. Repeated measurement data will be analyzed using repeated measures analysis of variance. Categorical variables will be presented as frequencies and proportions (%), with differences between groups evaluated through chi-square tests or Fisher’s exact tests.

Data analysis in this study will follow the intention-to-treat (ITT) principle, including all randomized participants regardless of adherence to the protocol or completion of the study (28). Missing data will be addressed under the missing-at-random assumption using sequential regression multiple imputation, which estimates missing values based on regression models that consider the relationships among all other variables (29). Furthermore, analyses will be performed on both the full analysis set (FAS) and the per-protocol set (PPS), with a *p*-value of less than 0.05 (two-tailed) is viewed as statistically significant.

### Ethical considerations

This clinical trial protocol received approval from the Cosmetic Ethics Committee of Shanghai Skin Disease Hospital (2025-03) and is registered with the International Traditional Medicine Clinical Trial Registry (ITMCTR2025001206). This study will be conducted in strict accordance with the ethical principles outlined in the Declaration of Helsinki. Any adverse events, protocol violations, or amendments to the study protocol or informed consent form will be reported to the Cosmetic Ethics Committee. Written informed consent will be collected from all participants prior to their involvement in this study, and all signed consent forms and completed CRF will be securely stored in a locked location, accessible only to authorized study personnel.

## Results

In this study, ethical approval was granted in March 2025, with registration completed in the International Traditional Medicine Clinical Trial Registry in June 2025. Recruitment of patient began in August 2025 and is anticipated to be concluded by June 2026. Data analysis is set to start in August 2026, submission of the preliminary trial results for peer review is anticipated by the end of December 2026.

## Discussion

This study aims to evaluate the efficacy of MSS combined with foundational therapies to alleviate symptoms of patients with mild-to-moderate AD, potentially providing a new therapeutic approach for the long term diseases management of AD. We hypothesize that MSS combined with foundational therapies will contribute to symptom relief in patients with mild-to-moderate AD.

AD not only manifests as pruritus, erythema, and dry, but also contribute to psychological issues such as sleep disturbances, anxiety, and depression (30–32). Moreover, compromised skin barrier function in AD patients increases their susceptibility to infections and makes them hypersensitive to environmental allergens and irritants (33). The long-term burden of AD not only affects patients’ physical health but can also have significant repercussions on their social and occupational lives (34, 35). While various treatment options are currently available, including corticosteroids, immunomodulators and biologics, some therapies still have side effects (36, 37), particularly in case of long-term usage (38, 39). Therefore, the development of novel effective, easily manageable and skin barrier repair treatment options remains an important clinical challenge.

For the treatment and long-term management of AD, skin barrier repair and maintenance are essential. Guidelines for AD treatment suggest that appropriate bathing can facilitate the repair of skin lesions (12, 13). Previous studies indicate that various bath solutions can efficiently restore skin barrier function and increase skin hydration (40, 41). A meta-analysis has demonstrated that traditional Chinese herbal bath solutions can effectively alleviate clinical pruritus (42). MSS, as a weakly acidic solution containing over seven core Chinese herbal components, helps inhibit inflammation and preserve skin barrier integrity (43). Numerous studies have demonstrated that the core Chinese herbal components can exert anti-inflammatory effects through three key pathways: inhibiting the release of inflammatory mediators, regulating immune cell activity, and reducing oxidative stress (44–46). By inhibit inflammation, repairing the barrier, alleviating symptoms, and expediting recovery, this herbal bath solution anticipated to substantially improve clinical outcomes in AD patients. Additionally, this study design includes foundational therapies (desonide cream for mild AD and triamcinolone acetonide cream for moderate AD) alongside rescue medication (levocetirizine tablets for severe pruritus, 1 tablet each day), ensuring that all AD patients receive standardized background care throughout the trial.

Results in this study are expected to provide new insights and approaches for the prevention and treatment of AD. To our knowledge, this study is the first clinical trial to assess the clinical efficacy and safety of a traditional Chinese medicine solution combined with foundational therapies in the treatment of mild-to moderate AD. The evaluation will be conducted through a multidimensional approach, encompassing assessments of skin lesion severity, intensity of pruritus, and overall quality of life. The results of this study will provide robust evidence for the incorporation of traditional Chinese herbal bath solutions into clinical practice as a supplementary treatment modality for AD, thereby effectively facilitate long-term management of AD. We are planned to disseminate our findings through publication in internationally peer-reviewed journals.

This study is subject to several limitations. First, patients recruitment is restricted to individuals with mild-to-moderate AD enrolled at the Shanghai Skin Disease Hospital, which enhancing internal validity while potentially limiting the generalizability of findings. Second, the protocol outlined a follow-up period lasting up to 4 months, which may result in patient dropout and lead to missing data and potentially impact the accuracy of the study’s findings. Thirdly, the study design, with a four-month follow-up, does not allow for evaluation of the long-term effects of MSS bath solution on AD recurrence rates. Therefore, it is essential to conduct a more relevant multi-center, randomized, placebo controlled clinical trials in the future.

## Conclusion

Findings in this study are expected to provide evidence for the incorporation of traditional Chinese herbal bath solutions into the comprehensive management of AD, and ultimately alleviate disease burden associated with AD.
